# The genome sequence of the Annual Mercury, *Mercurialis annua *L., 1753 (Euphorbiaceae)

**DOI:** 10.12688/wellcomeopenres.21004.1

**Published:** 2024-03-01

**Authors:** Maarten J. M. Christenhusz, John R. Pannell, Alex D. Twyford, Royal Botanic Gardens Kew Genome Acquisition Lab, Plant Genome Sizing collective, Darwin Tree of Life Barcoding collective, Wellcome Sanger Institute Tree of Life programme, Wellcome Sanger Institute Scientific Operations: DNA Pipelines collective, Tree of Life Core Informatics collective, Darwin Tree of Life Consortium

**Affiliations:** 1 Royal Botanic Gardens Kew, Richmond, England, UK; 2 Curtin University, Perth, Western Australia, Australia; 3 Department of Ecology and Evolution, University of Lausanne, Lausanne, Switzerland; 4 The University of Edinburgh, Edinburgh, Scotland, UK; 5 Royal Botanic Garden Edinburgh, Edinburgh, Scotland, UK

**Keywords:** Mercurialis annua, Annual Mercury, genome sequence, chromosomal, Malpighiales

## Abstract

We present a genome assembly from a diploid female *Mercurialis annua* (the Annual Mercury; Tracheophyta; Magnoliopsida; Malpighiales; Euphorbiaceae). The genome sequence is 453.2 megabases in span. Most of the assembly is scaffolded into 8 chromosomal pseudomolecules, including the X chromosome. The organelle genomes have also been assembled, and the mitochondrial genome is 435.28 kilobases in length, while the plastid genome is 169.65 kilobases in length.

## Species taxonomy

Eukaryota; Viridiplantae; Streptophyta; Streptophytina; Embryophyta; Tracheophyta; Euphyllophyta; Spermatophyta; Magnoliopsida; Mesangiospermae; eudicotyledons; Gunneridae; Pentapetalae; rosids; fabids; Malpighiales; Euphorbiaceae; Acalyphoideae; Acalypheae; *Mercurialis*; *Mercurialis annua* L., 1753 (NCBI:txid3986).

## Background

The Annual Mercury *Mercurialis annua* is a widespread species, native to Europe, north Africa and western Asia. It is an ancient introduction (archaeophyte) in Britain and Ireland, where it is most abundant in England, decreasing in abundance to the north, being generally rare in Scotland ( Stace, 2010). The species is a wind-pollinated annual, found predominantly in disturbed habitats such as roadsides and gardens. It can be distinguished from the native congener *M. perennis* based on a range of traits such as its lack of a perennating rhizome, more frequent branching and paler green colour, as well as its distinct ecological preference for less shady sites.

When one of us (JRP) began his investigations as a graduate student into the ecological and genetic reasons for the evolution and maintenance of dioecy, he adopted the rare case of dioecy in the annual species *M. annua* as a model. *M. annua* had featured in an early woodblock published by Linnaeus (1749) that drew attention to the sexuality of plants and the transfer of pollen from males to females. It was also studied by Kuhn (1939) in investigations of the meaning of sex-ratio variation and in early work by Westergaard (1958) on mechanisms of sex determination in plants. In 1963, a French student Bernard Durand published a PhD thesis as a monograph on the biosystematics and cytogenetics of *M. annua*, which, he revealed, is a complex of several polyploid lineages that vary in their sexual system ( Durand, 1963). Whereas diploid *M. annua* is dioecious, higher ploidy levels are variously monoecious and androdioecious (the co-occurrence in a population of males and functional hermaphrodites – in this case, males and monoecious individuals) ( Durand, 1963; Durand & Durand, 1992). Androdioecy is a very rare sexual system that was still completely unrecognised for any plant species ( Charlesworth, 1984) until 1990 ( Liston *et al.*, 1990) – Durand’s (1963) monograph had been overlooked ( Pannell, 1997). Over the last three decades, however, the *M. annua* species complex has proven a rich model to study not only evolutionary transitions between sexual systems (reviewed in Pannell *et al*., 2008) and ploidy levels (reviewed in Pannell *et al*., 2004), but also the ecological genetics and genomic implications of metapopulations and range expansions ( González-Martínez *et al.*, 2017; Obbard *et al.*, 2006; Pujol *et al.*, 2009; Pujol & Pannell, 2008).

Work on the sex chromosomes of *M. annua* prompted the first genome assembly for the species ( Veltsos *et al.*, 2018). This assembly was based on Illumina short reads and low-coverage early generation PacBio long reads for scaffolding of a male *M. annua*. The resulting assembly was highly fragmented (720,537 scaffolds, scaffold N50 of 6,398 bp) and had a limited completeness based on BUSCO scores (76.1% of complete BUSCOs). Despite its relatively low contiguity, this first assembly proved useful for studies of the genomic implications of the species’ range expansion in Europe ( González-Martínez *et al.*, 2017) and the evolution of sex chromosomes in both diploid and polyploid *M. annua* ( Gerchen *et al.*, 2022; Toups *et al.*, 2022; Veltsos *et al.*, 2018, Veltsos *et al.*, 2019).

A new reference genome of *M. annua* has now been sequenced as part of the Darwin Tree of Life Project. Here we present the chromosomally complete genome sequence for this species, based on one female specimen collected from the Royal Botanic Gardens, Kew. This new genome has a substantially higher contiguity and completeness than earlier assemblies, and is already providing a timely and much-needed resource. The genome is being used in the investigation of the spectacularly rapid breakdown of dioecy observed in replicate populations from which males were initially experimentally removed and in which the ‘leaky’ production of male flowers by females has been amplified many fold by frequency-dependent selection on the population sex allocation ( Cossard *et al.*, 2021). It will also strengthen ongoing work on the comparative genomics of sex chromosomes across the genus in the context of genome duplication, hybridisation and the introgression of the Y chromosome between distantly related species. Finally, it will be valuable for ongoing ecological genomic analyses of the impact of admixture among populations on population dynamics and local adaptation at and beyond species range margins. We plan to supplement this genome assembly from an XX female with a genome from a male, to understand differences in structure and gene content between X and Y chromosomes.

## Genome sequence report

**Figure 1.**
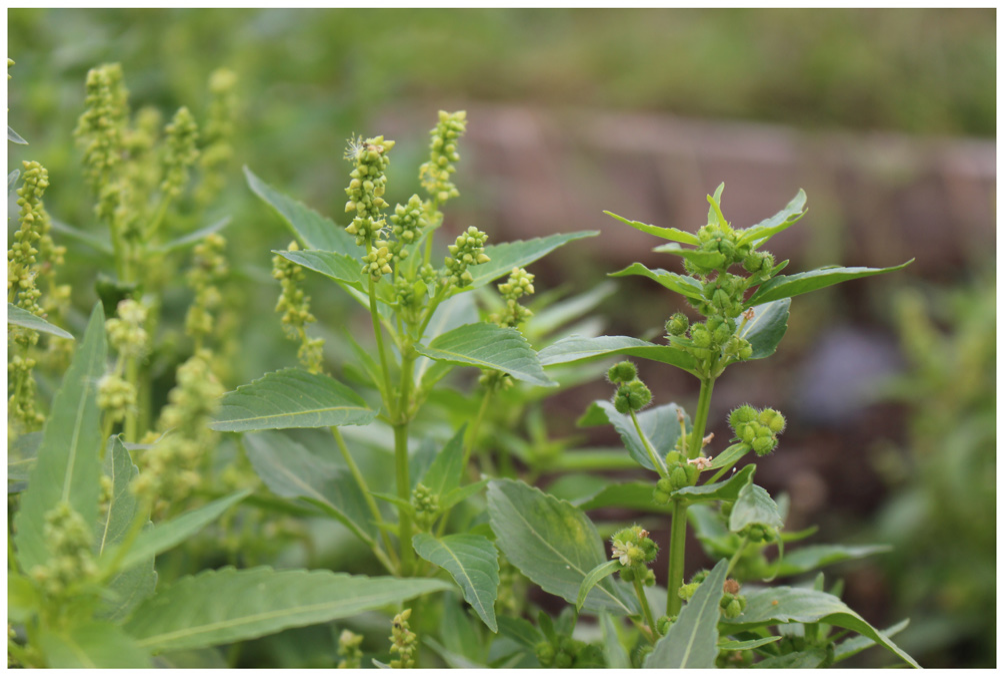
Photograph of male and female *Mercurialis annua*.

The genome was sequenced from one female *Mercurialis annua* ( Figure 1) collected from Royal Botanic Gardens, Kew (51.48, –0.30). Using flow cytometry, the genome size (1C-value) was estimated to be 0.71 pg, equivalent to 690 Mb. A total of 58-fold coverage in Pacific Biosciences single-molecule HiFi long reads and 102-fold coverage in 10X Genomics read clouds were generated. Primary assembly contigs were scaffolded with chromosome conformation Hi-C data. Manual assembly curation corrected 12 missing joins or mis-joins and removed 7 haplotypic duplications, reducing the assembly length by 0.67% and the scaffold number by 1.52%, and decreasing the scaffold N50 by 7.45%.

**Figure 2.**
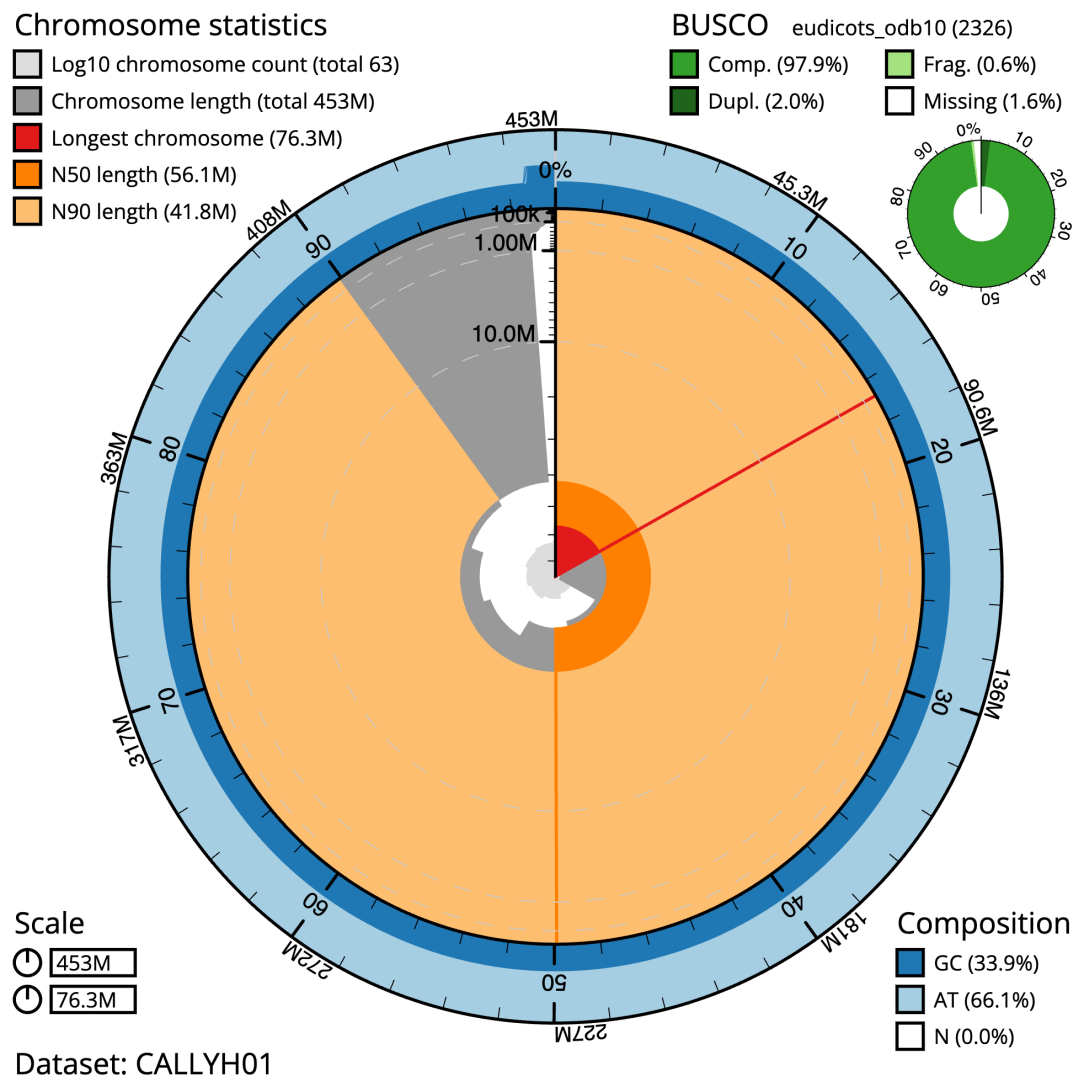
Genome assembly of *Mercurialis annua*, ddMerAnnu1.2: metrics. The BlobToolKit Snailplot shows N50 metrics and BUSCO gene completeness. The main plot is divided into 1,000 size-ordered bins around the circumference with each bin representing 0.1% of the 453,168,992 bp assembly. The distribution of scaffold lengths is shown in dark grey with the plot radius scaled to the longest scaffold present in the assembly (76,280,018 bp, shown in red). Orange and pale-orange arcs show the N50 and N90 scaffold lengths (56,051,264 and 41,830,924 bp), respectively. The pale grey spiral shows the cumulative scaffold count on a log scale with white scale lines showing successive orders of magnitude. The blue and pale-blue area around the outside of the plot shows the distribution of GC, AT and N percentages in the same bins as the inner plot. A summary of complete, fragmented, duplicated and missing BUSCO genes in the eudicots_odb10 set is shown in the top right. An interactive version of this figure is available at https://blobtoolkit.genomehubs.org/view/ddMerAnnu1.1/dataset/CALLYH01/snail.

**Figure 3.**
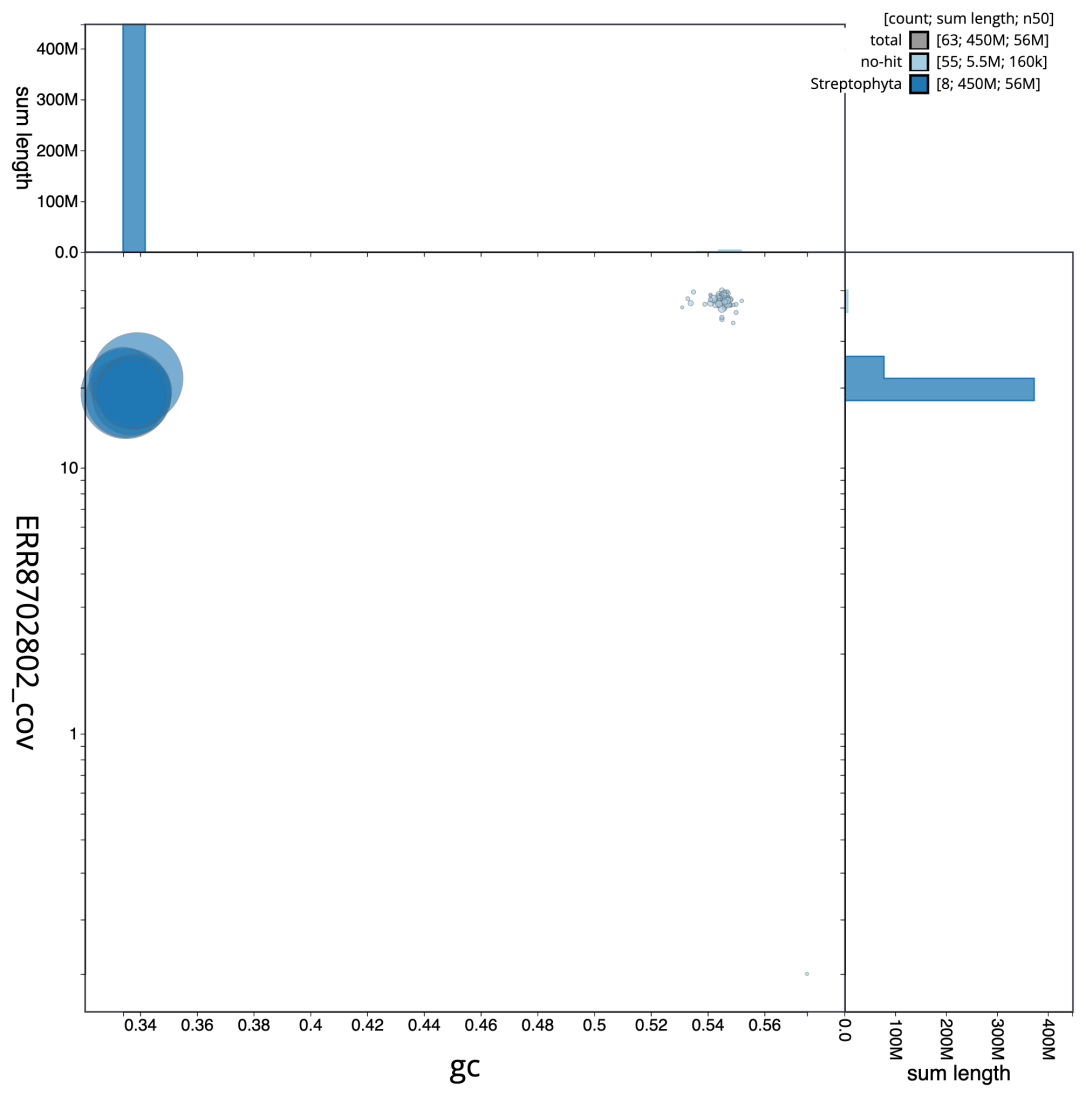
Genome assembly of *Mercurialis annua*, ddMerAnnu1.2: BlobToolKit GC-coverage plot. Scaffolds are coloured by phylum. Circles are sized in proportion to scaffold length. Histograms show the distribution of scaffold length sum along each axis. An interactive version of this figure is available at https://blobtoolkit.genomehubs.org/view/ddMerAnnu1.1/dataset/CALLYH01/blob.

**Figure 4.**
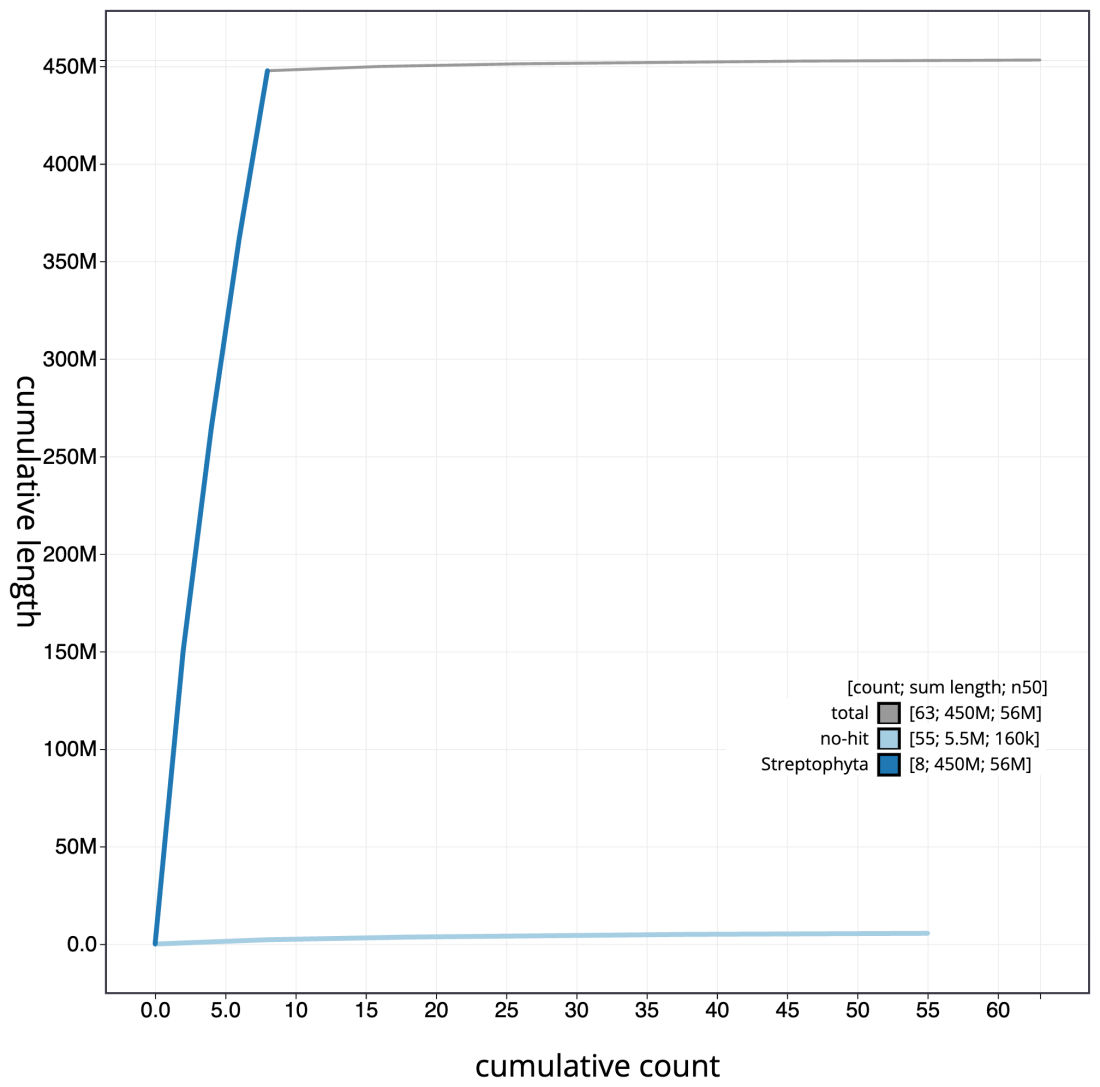
Genome assembly of *Mercurialis annua*, ddMerAnnu1.2: BlobToolKit cumulative sequence plot. The grey line shows cumulative length for all scaffolds. Coloured lines show cumulative lengths of scaffolds assigned to each phylum using the buscogenes taxrule. An interactive version of this figure is available at https://blobtoolkit.genomehubs.org/view/ddMerAnnu1.1/dataset/CALLYH01/cumulative.

**Figure 5.**
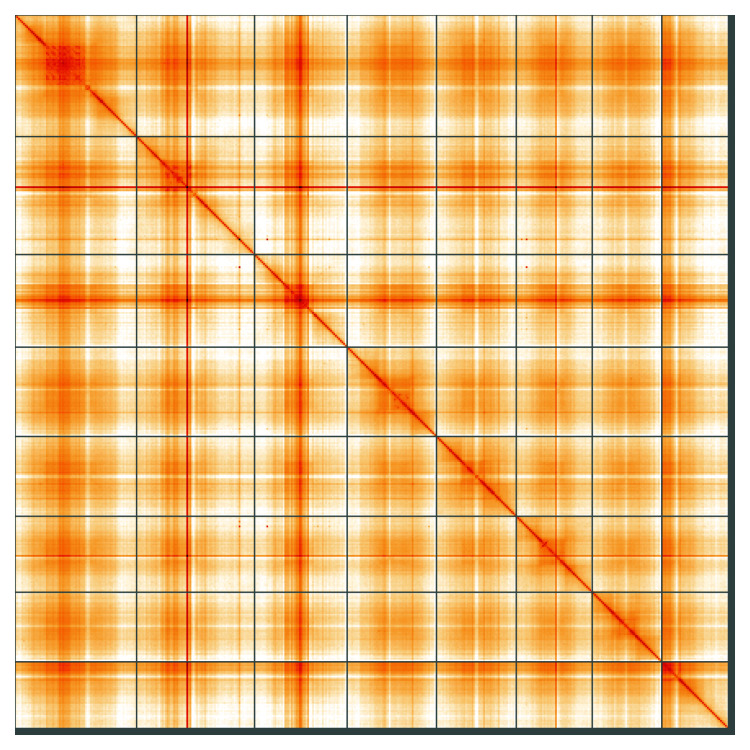
Genome assembly of *Mercurialis annua*, ddMerAnnu1.2: Hi-C contact map of the ddMerAnnu1.2 assembly, visualised using HiGlass. Chromosomes are shown in order of size from left to right and top to bottom. An interactive version of this figure may be viewed at https://genome-note-higlass.tol.sanger.ac.uk/l/?d=Q-UX07cRTeCFRydfKzUNKA.

The final assembly has a total length of 453.2 Mb in 63 sequence scaffolds with a scaffold N50 of 56.1 Mb ( Table 1). The snailplot in Figure 2 provides a summary of the assembly statistics, while the distribution of assembly scaffolds on GC proportion and coverage is shown in Figure 3. The cumulative assembly plot in Figure 4 shows curves for subsets of scaffolds assigned to different phyla. Most (99.86%) of the assembly sequence was assigned to 8 chromosomal-level scaffolds. Chromosome assignment for this assembly is based on the genetic map produced by ( Veltsos *et al.*, 2019). Since the sex chromosomes are homomorphic and have recombined over an extensive autosomal region, they were identified as a single linkage group, therefore, for this diploid female specimen, X has been assigned LG1-X ( Figure 5; Table 2). While not fully phased, the assembly deposited is of one haplotype. Contigs corresponding to the second haplotype have also been deposited. The mitochondrial and plastid genomes were also assembled and deposited.

**Table 1.** Genome data for *Mercurialis annua*, ddMerAnnu1.2.

Project accession data		
Assembly identifier	ddMerAnnu1.2	
Species	*Mercurialis annua*	
Specimen	ddMerAnnu1	
NCBI taxonomy ID	3986	
BioProject	PRJEB50972	
BioSample ID	SAMEA7522176	
Isolate information	ddMerAnnu1, female: leaf (DNA sequencing, Hi-C scaffolding, RNA sequencing)	
Assembly metrics *		*Benchmark*
Consensus quality (QV)	52.8	≥ *50*
*k*-mer completeness	99.98%	≥ *95%*
BUSCO **	C:97.9%[S:95.9%,D:2.0%], F:0.6%,M:1.6%,n:2,326	*C* ≥ *95%*
Percentage of assembly mapped to chromosomes	99.86%	≥ *95%*
Sex chromosomes	X assigned to linkage group 1	*localised homologous pairs*
Organelles	Mitochondrial and plastid genomes	*complete single alleles*
Raw data accessions		
PacificBiosciences SEQUEL II	ERR8705877, ERR8705876	
10X Genomics Illumina	ERR8702801, ERR8702802, ERR8702803, ERR8702804, ERR8702806, ERR8702807, ERR8702808, ERR8702805	
Hi-C Illumina	ERR8702809	
PolyA RNA-Seq Illumina	ERR10123682	
Genome assembly		
Assembly accession	GCA_937616625.2	
*Accession of alternate haplotype*	GCA_937609235.2	
Span (Mb)	453.2	
Number of contigs	78	
Contig N50 length (Mb)	35.6	
Number of scaffolds	63	
Scaffold N50 length (Mb)	56.1	
Longest scaffold (Mb)	76.3	

* Assembly metric benchmarks are adapted from column VGP-2020 of “Table 1: Proposed standards and metrics for defining genome assembly quality” from Rhie *et al.* (2021).

* BUSCO scores based on the eudicots_odb10 BUSCO set using v5.3.2. C = complete [S = single copy, D = duplicated], F = fragmented, M = missing, n = number of orthologues in comparison. A full set of BUSCO scores is available at https://blobtoolkit.genomehubs.org/view/ddMerAnnu1.1/dataset/CALLYH01/busco.

**Table 2.** Chromosomal pseudomolecules in the genome assembly of *Mercurialis annua*, ddMerAnnu1.

INSDC accession	Chromosome	Size (Mb)	GC%
OW569319.1	LG1-X	74.05	33.5
OW569313.1	LG2	58.1	33.5
OW569312.1	LG3	76.28	33.9
OW569317.1	LG4	41.83	33.4
OW569314.1	LG5	56.05	33.7
OW569316.1	LG6	47.72	33.8
OW569315.1	LG7	50.03	33.8
OW569318.1	LG8	43.61	33.7
OX359233.1	MT	0.44	44.1
OX359234.1	Pltd	0.17	35.7

The estimated Quality Value (QV) of the final assembly is 52.8 with *k*-mer completeness of 99.98%, and the assembly has a BUSCO v5.3.2 completeness of 97.9% (single = 95.9%, duplicated = 2.0%), using the eudicots_odb10 reference set ( *n* = 2,326).

Metadata for specimens, spectral estimates, sequencing runs, contaminants and pre-curation assembly statistics can be found at https://links.tol.sanger.ac.uk/species/3986.

## Methods

### Sample acquisition, genome size estimation and nucleic acid extraction

A female *Mercurialis annua* (specimen ID KDTOL10055, ToLID ddMerAnnu1) was collected from Royal Botanic Gardens Kew, Surrey, UK at a drain between White Peaks and the Lower Nursery (latitude 51.48, longitude –0.30) on 2020-08-26. The specimen was picked by hand from weedy vegetation by Maarten Christenhusz (Royal Botanic Gardens, Kew). The specimen was also formally identified by Maarten Christenhusz, and then preserved by freezing at –80°C.

The genome size was estimated by flow cytometry using the fluorochrome propidium iodide and following the ‘one-step’ method as outlined in Pellicer *et al*. (2021). Specifically for this species, CyStain™ PI OxProtect Staining Buffer (cat. No. 05-5027; Sysmex UK Ltd.) was used for isolation of nuclei, and the internal calibration standard was *Petroselinum crispum* ‘Champion Moss Curled’ with an assumed 1C-value of 2,200 Mb ( Obermayer *et al.*, 2002).

DNA was extracted at the Tree of Life laboratory, Wellcome Sanger Institute (WSI). The ddMerAnnu1 sample was weighed and dissected on dry ice with tissue set aside for Hi-C sequencing. Leaf tissue was cryogenically disrupted to a fine powder using a Covaris cryoPREP Automated Dry Pulveriser, receiving multiple impacts. High molecular weight (HMW) DNA was extracted using the Qiagen MagAttract HMW DNA extraction kit. Low molecular weight DNA was removed from a 20 ng aliquot of extracted DNA using the 0.8X AMpure XP purification kit prior to 10X Chromium sequencing; a minimum of 50 ng DNA was submitted for 10X sequencing. HMW DNA was sheared into an average fragment size of 12–20 kb in a Megaruptor 3 system with speed setting 30. Sheared DNA was purified by solid-phase reversible immobilisation using AMPure PB beads with a 1.8X ratio of beads to sample to remove the shorter fragments and concentrate the DNA sample. The concentration of the sheared and purified DNA was assessed using a Nanodrop spectrophotometer and Qubit Fluorometer and Qubit dsDNA High Sensitivity Assay kit. Fragment size distribution was evaluated by running the sample on the FemtoPulse system.

RNA was extracted from leaf tissue of ddMerAnnu1 in the Tree of Life Laboratory at the WSI using TRIzol, according to the manufacturer’s instructions. RNA was then eluted in 50 μl RNAse-free water and its concentration assessed using a Nanodrop spectrophotometer and Qubit Fluorometer using the Qubit RNA Broad-Range (BR) Assay kit. Analysis of the integrity of the RNA was done using an Agilent RNA 6000 Pico Kit and Eukaryotic Total RNA assay.

### Sequencing

Pacific Biosciences HiFi circular consensus and 10X Genomics read cloud DNA sequencing libraries were constructed according to the manufacturers’ instructions. Poly(A) RNA-Seq libraries were constructed using the NEB Ultra II RNA Library Prep kit. DNA and RNA sequencing was performed by the Scientific Operations core at the WSI on Pacific Biosciences SEQUEL II (HiFi), Illumina NovaSeq 6000 (RNA-Seq) and Illumina NovaSeq 6000 (10X) instruments. Hi-C data were also generated from leaf tissue of ddMerAnnu1 using the Arima2 kit and sequenced on the Illumina NovaSeq 6000 instrument.

### Genome assembly, curation and evaluation

Assembly was carried out with Hifiasm ( Cheng *et al.*, 2021) and haplotypic duplication was identified and removed with purge_dups ( Guan *et al.*, 2020). One round of polishing was performed by aligning 10X Genomics read data to the assembly with Long Ranger ALIGN, calling variants with FreeBayes ( Garrison & Marth, 2012). The assembly was then scaffolded with Hi-C data ( Rao *et al.*, 2014) using YaHS ( Zhou *et al.*, 2023). The assembly was checked for contamination and corrected using the gEVAL system ( Chow *et al.*, 2016) as described previously ( Howe *et al.*, 2021). Manual curation was performed using gEVAL, HiGlass ( Kerpedjiev *et al.*, 2018) and Pretext ( Harry, 2022). The mitochondrial genome was assembled using MitoHiFi ( Uliano-Silva *et al.*, 2022), which runs MitoFinder ( Allio *et al.*, 2020) or MITOS ( Bernt *et al.*, 2013) and uses these annotations to select the final mitochondrial contig and to ensure the general quality of the sequence. The mitochondrial and plastid genomes were assembled using MBG ( Rautiainen & Marschall, 2021) from PacBio HiFi reads mapping to related genomes. A representative circular sequence was selected for each from the graph based on read coverage.

A Hi-C map for the final assembly was produced using bwa-mem2 ( Vasimuddin *et al.*, 2019) in the Cooler file format ( Abdennur & Mirny, 2020). To assess the assembly metrics, the *k*-mer completeness and QV consensus quality values were calculated in Merqury ( Rhie *et al.*, 2020). This work was done using Nextflow ( Di Tommaso *et al.*, 2017) DSL2 pipelines “sanger-tol/readmapping” ( Surana *et al.*, 2023a) and “sanger-tol/genomenote” ( Surana *et al.*, 2023b). The genome was analysed within the BlobToolKit environment ( Challis *et al.*, 2020) and BUSCO scores ( Manni *et al.*, 2021; Simão *et al.*, 2015) were calculated.

Table 3 contains a list of relevant software tool versions and sources.

**Table 3.** Software tools: versions and sources.

Software tool	Version	Source
BlobToolKit	3.4.0	https://github.com/blobtoolkit/blobtoolkit
BUSCO	5.3.2	https://gitlab.com/ezlab/busco
FreeBayes	1.3.1-17-gaa2ace8	https://github.com/freebayes/freebayes
gEVAL	N/A	https://geval.org.uk/
Hifiasm	0.15.3	https://github.com/chhylp123/hifiasm
HiGlass	1.11.6	https://github.com/higlass/higlass
Long Ranger ALIGN	2.2.2	https://support.10xgenomics.com/genome-exome/ software/pipelines/latest/advanced/other-pipelines
Merqury	MerquryFK	https://github.com/thegenemyers/MERQURY.FK
MBG	_	https://github.com/maickrau/MBG
PretextView	0.2	https://github.com/wtsi-hpag/PretextView
purge_dups	1.2.3	https://github.com/dfguan/purge_dups
sanger-tol/genomenote	v1.0	https://github.com/sanger-tol/genomenote
sanger-tol/readmapping	1.1.0	https://github.com/sanger-tol/readmapping/tree/1.1.0
YaHS	1.0	https://github.com/c-zhou/yahs

### Wellcome Sanger Institute – Legal and Governance

The materials that have contributed to this genome note have been supplied by a Darwin Tree of Life Partner. The submission of materials by a Darwin Tree of Life Partner is subject to the ‘Darwin Tree of Life Project Sampling Code of Practice’, which can be found in full on the Darwin Tree of Life website here. By agreeing with and signing up to the Sampling Code of Practice, the Darwin Tree of Life Partner agrees they will meet the legal and ethical requirements and standards set out within this document in respect of all samples acquired for, and supplied to, the Darwin Tree of Life Project.

Further, the Wellcome Sanger Institute employs a process whereby due diligence is carried out proportionate to the nature of the materials themselves, and the circumstances under which they have been/are to be collected and provided for use. The purpose of this is to address and mitigate any potential legal and/or ethical implications of receipt and use of the materials as part of the research project, and to ensure that in doing so we align with best practice wherever possible. The overarching areas of consideration are:

•   Ethical review of provenance and sourcing of the material

•   Legality of collection, transfer and use (national and international)

Each transfer of samples is further undertaken according to a Research Collaboration Agreement or Material Transfer Agreement entered into by the Darwin Tree of Life Partner, Genome Research Limited (operating as the Wellcome Sanger Institute), and in some circumstances other Darwin Tree of Life collaborators.

## Data Availability

European Nucleotide Archive: *Mercurialis annua*. Accession number PRJEB50972; https://identifiers.org/ena.embl/PRJEB50972 ( Wellcome Sanger Institute, 2022). The genome sequence is released openly for reuse. The *Mercurialis annua* genome sequencing initiative is part of the Darwin Tree of Life (DToL) project. All raw sequence data and the assembly have been deposited in INSDC databases. The genome will be annotated using available RNA-Seq data and presented through the Ensembl pipeline at the European Bioinformatics Institute. Raw data and assembly accession identifiers are reported in Table 1.

## Author information

Members of the Royal Botanic Gardens Kew Genome Acquisition Lab are listed here: https://doi.org/10.5281/zenodo.4786680.

Members of the Darwin Tree of Life Barcoding collective are listed here: https://doi.org/10.5281/zenodo.4893703.

Members of the Plant Genome Sizing collective are listed here: https://doi.org/10.5281/zenodo.7994306.

Members of the Wellcome Sanger Institute Tree of Life programme are listed here: https://doi.org/10.5281/zenodo.4783585.

Members of Wellcome Sanger Institute Scientific Operations: DNA Pipelines collective are listed here: https://doi.org/10.5281/zenodo.4790455.

Members of the Tree of Life Core Informatics collective are listed here: https://doi.org/10.5281/zenodo.5013541.

Members of the Darwin Tree of Life Consortium are listed here: https://doi.org/10.5281/zenodo.4783558.
