# Supplementary material for: The genome sequence of the Annual Mercury, Mercurialis annua L., 1753 (Euphorbiaceae)
Source: Wellcome Open Res. 2024 Mar 1;9:102. [Version 1] doi: 10.12688/wellcomeopenres.21004.1 (PMC11157195; doi:10.12688/wellcomeopenres.21004.1)
Supplement: Peer review history

# Reviewer report: Reviewer response for version 1

**DOI:** 10.21956/wellcomeopenres.23239.r82768

**Published:** 2024-06-06

**Authors:** Yoshinori Fukasawa

**Affiliations:** 1 Center for Center for Bioscience Research and Education, Utsunomiya University, Tochigi, Japan

This study presents a novel genome assembly for the Annual Mercury, *Mercurialis annua*, a widespread plant species renowned for its complex sexual system. The assembly, generated using PacBio HiFi reads and supplemented with 10X Genomics linked reads, was scaffolded using Hi-C data.

 The resulting assembly represents a substantial improvement over prior assemblies in terms of contiguity and completeness, as evidenced by high BUSCO scores and consensus quality values determined using Merqury.

 The assembled genome spans 453.2 Mb and is predominantly scaffolded into eight chromosomal pseudomolecules, including the X chromosome. The mitochondrial and plastid genomes were also successfully assembled.

 This high-quality genome assembly serves as a valuable reference for *Mercurialis annua* and meets current technical standards in the field. The assembly exhibits significantly improved contiguity and completeness compared to previous efforts, making it an invaluable resource for future research. It comprehensively encompasses the entire genetic sequence, including organelle genomes.

 Minor points should be addressed:

 1 - Mitochondrial Genome Assembly: The authors should explicitly state which method was primarily used for the mitochondrial genome assembly, either MitoHiFi or MBG, and provide a clear rationale for their choice. The current description creates ambiguity and necessitates further clarification.

 2 - Origin of K-mers: The source of the k-mers used in Merqury should be clearly stated. This could be addressed by specifying whether they were derived from HiFi reads, 10X linked reads, or a combination of both.

 3 - Figure 5: Labels for both x- and y-axes, along with clear identification of each chromosome, should be included in Figure 5 to improve its clarity and interpretability.

 4 - High-Level Statistics: The authors could consider including additional high-level statistics, such as the presence or absence of telomeres and centromeres, to provide a more comprehensive overview of the assembly's quality and completeness for this level of contiguity. These statistics would be particularly relevant for readers interested in the structural organization of the *Mercurialis annua* genome.

Are sufficient details of methods and materials provided to allow replication by others?

Partly

Is the rationale for creating the dataset(s) clearly described?

Yes

Are the datasets clearly presented in a useable and accessible format?

Yes

Are the protocols appropriate and is the work technically sound?

Yes

Reviewer Expertise:

Plant genomics and bioinformatics

I confirm that I have read this submission and believe that I have an appropriate level of expertise to confirm that it is of an acceptable scientific standard.

# Reviewer report: Reviewer response for version 1

**DOI:** 10.21956/wellcomeopenres.23239.r84817

**Published:** 2024-06-06

**Authors:** Hoang Dang Khoa Do

**Affiliations:** 1 NTT Hi-Tech Institute, Nguyen Tat Thanh University, Ho Chi Minh City, Ho Chi Minh, Vietnam

The authors reported a procedure for sequencing and assembling the nuclear genome of Mercurialis annua. Although the complete sequences of chromosomes were not reported, the 8 chromosomal pseudomolecules, including the X chromosome provided new insights into genomic data of M. annua in comparison to previous studies. The X chromosome sequence is a useful source for further comparative genomic studies about sex chromosomes in M. annua. Additionally, the complete chloroplast and mitochondrial genomes of M. annua were successfully sequenced. However, the authors might characterize the features of these organelle genomes which are essential data for exploring the evolutionary history of M. annua.

Are sufficient details of methods and materials provided to allow replication by others?

Yes

Is the rationale for creating the dataset(s) clearly described?

Yes

Are the datasets clearly presented in a useable and accessible format?

Yes

Are the protocols appropriate and is the work technically sound?

Yes

Reviewer Expertise:

Plant Genomes, Plant Systematics, Genomic Evolution.

I confirm that I have read this submission and believe that I have an appropriate level of expertise to confirm that it is of an acceptable scientific standard.
